# Underreporting of methicillin-resistant *Staphylococcus aureus* (MRSA) carriage by surgeons in the operating room planning software

**DOI:** 10.1186/1753-6561-5-S6-P174

**Published:** 2011-06-29

**Authors:** C Bandelier, G Zanetti, L Senn

**Affiliations:** 1 CHUV, Lausanne, Switzerland

## Introduction / objectives

MRSA carriage should be notified to operating room (OR) teams in order to guarantee continuity of contact isolation precautions, planning of MRSA carriers after non-carriers, and adequate antibiotic prophylaxis if indicated. The objective of our study was to assess the proportion of known MRSA carriers notified in the OR software.

## Methods

In our hospital, the surgeon in charge is responsible for notifying patients who carry multiresistant bacteria in the OR software at time of intervention planning. We merged the OR database with the MRSA database of our laboratory. All surgical interventions (10 surgical specialties) conducted between January and December 2010 were analyzed.

## Results

11’701 interventions were analyzed. We identified 584 (5%) interventions in known MRSA carriers. Only 318/584 (54.5%) MRSA carriers were notified as such by the surgeon in charge in the OR planning software. This reporting rate varied from 23% to 72% depending on the surgical specialties. It increased significantly with the mean weekly prevalence of MRSA carriers on each specialty ward in a weighted regression model (regression coefficient: 2.9, p=0.046).

## Conclusion

Surgical interventions in MRSA carriers represented 5% of all interventions in our hospital. Pre-operative MRSA carriage was notified to the OR team in only 54.5% of cases, which may increase the risk of transmission and prejudice adequate antibiotic prophylaxis. Notification rates were associated with the burden of MRSA cases on the wards, which may highlight an insufficient awareness of the problem among surgeons. Beside education efforts, an automated transfer of MRSA information to the OR database should be implemented.

## Disclosure of interest

None declared.

